# Metabolomic and lipidomic studies on the intervention of taurochenodeoxycholic acid in mice with hyperlipidemia

**DOI:** 10.3389/fphar.2023.1255931

**Published:** 2023-11-15

**Authors:** Na Cui, Wensen Zhang, Fazhi Su, Zhihong Zhang, Biao Li, Donghui Peng, Yanping Sun, Yuanning Zeng, Bingyou Yang, Haixue Kuang, Qiuhong Wang

**Affiliations:** ^1^ Key Laboratory of Basic and Application Research of Beiyao, Ministry of Education, Heilongjiang University of Chinese Medicine, Harbin, China; ^2^ School of Chinese Materia Medica, Guangdong Engineering Technology Research Center for Standardized Processing of Chinese Materia Medica Guangdong Pharmaceutical University, Guangdong, China

**Keywords:** taurochenodeoxycholic acid, hyperlipidemia, metabolomics, lipidomics, glycerophospholipid metabolism

## Abstract

Bile acids are the main component of animal bile and are directly involved in the metabolic process of lipids in vivo. Taurochenodeoxycholic acid (TCDCA) is the primary biologically active substance in bile acids and has biological functions such as antioxidant, antipyretic, anti-inflammatory, and analgesic activities and improves immunity. In the present study, we assessed the impact of TCDCA on hyperlipidemia development in mouse models. Mice were fed a high-fat diet (HFD) to induce hyperlipidemia and orally administered different doses of TCDCA orally for 30 days. Then, indicators such as triglyceride (TG), total cholesterol (TC), low-density lipoprotein cholesterol (LDL-C), and high-density lipoprotein cholesterol (HDL-C) in mice were detected. Using HE and ORO staining techniques, the morphology of the mice’s liver tissue was detected. Based on metabolomic and lipidomic analyses, we determined the mechanism of TCDCA in treating hyperlipidemia. The results showed that TCDCA had a significant ameliorating effect on dietary hyperlipidemia. In addition, it exerted therapeutic effects through glycerophospholipid metabolism.

## 1 Introduction

With the aging of the global population and improving living standards, the number of people suffering from metabolic diseases is gradually increasing. Common conditions include hyperlipidemia, diabetes mellitus, and coronary heart disease (Jia et al., 2021). Among them, hyperlipidemia has many pathogenic factors. Excessive intake of fat (Sumiyoshi et al., 2006), abnormal lipoprotein synthesis, and metabolism can lead to dyslipidemia (Kaysen, 1992). Dyslipidemia can lead to lipid metabolism disorders and is the main pathogenic factor of hyperlipidemia (Koopal et al., 2017). Since various metabolic enzymes are secreted by the liver, improper functioning of the liver results in fat buildup that can easily cause excessive lipid synthesis and secretion, leading to hyperlipidemia over time (Michels et al., 2022).

Taurochenodeoxycholic acid (TCDCA) is a bile acid derivative that combines two molecules of taurine and chenodeoxycholic acid and is found in high concentrations in the bile of poultry and livestock (Zhong et al., 2021; Nguyen and Bouscarel, 2008). It is clear that bile acids facilitate digestion, absorption, and excretion of dietary lipids and interact with numerous cellular signaling pathways (Liu et al., 2011). TCDCA is also used as a nutritional fortifier added to foods. TCDCA is one of the main active ingredients of bear bile powder (Shi et al., 2017). It has been shown that TCDCA is used to treat diseases such as astrocytic neuroinflammation (Xu et al., 2023a), pulmonary fibrosis (Zhou et al., 2013), and gastric cancer (Zhang et al., 2022), with inhibitory effects on both acute and chronic inflammation as well as modulating immune responses (Li et al., 2019; Bao et al., 2021). Recent studies have reported that TCDCA can reduce intestinal fat transport and treat non-alcoholic fatty liver disease in mice (Wang et al., 2018). Moreover, natural products play a crucial role in treating metabolic disorders in human diseases.

In this study, we focused on the effect of TCDCA on dietary HFD. TCDCA improves lipid metabolism abnormalities by altering the glycerophospholipid metabolism pathway and ameliorates HFD-associated dyslipidemia and hepatic injury. TCDCA is expected to be a promising lipid-lowering active ingredient, which opens new avenues for studying its therapeutic use in treating metabolic diseases. In this regard, TCDCA has become the current research hotspot.

## 2 Materials and methods

### 2.1 Animals and ethics statement

This study followed the Chinese Guide for the Care and Use of Laboratory Animals guidelines. All animal experiments were approved by the Laboratory Animal Ethics Committee of Guangdong Medical Laboratory Animal Center (Approval No. C202211-1). Forty-eight adult male C57BL/6 mice were obtained from Guangdong Medical Laboratory Animal Center (Guangdong Province, China, SCXK (Guangdong) 2022–0002.). The mice were housed in a standard room (12/12-h light/dark cycle, 23°C ± 2°C; relative humidity: 55% ± 2%) with food and water provided *ad libitum*. Mice were randomly divided into six groups: control group, model group, fenofibrate group, high-dose group (TCDCA-H), medium-dose group (TCDCA-M), and low-dose group (TCDCA-L).

### 2.2 Chemicals and reagents

TCDCA was purchased from Macklin (Shanghai, China, batch number: C10584895). HFD was purchased from Jiangsu Synergistic Pharmaceutical Bioengineering Co. AST, ALT, TC, TG, LDL-C, and HDL-C kits were purchased from Nanjing Jiancheng Biotechnology Co., Ltd. (Nanjing City, Jiangsu Province, China, batch number: 20220509). Hematoxylin and Eosin (HE) and Oil Red O (ORO) staining kits were purchased from Servicebio (Wuhan City, Hubei Province, China; batch numbers: G1003 and G1015). Mass spectrometry-grade formic acid and acetonitrile were purchased from Thermo Fisher (Thermo Fisher, United States, batch numbers: 205178 and 205187).

### 2.3 Establishment of the hyperlipidemia model

The control group was fed ordinary feed (containing 18.5% protein, 4.6% fat, 58.9% carbohydrate, 3.2% crude fiber, 6.8% ash, 1.28% calcium, and 0.92 phosphorus), and the other groups were fed HFD for 30 days (containing 22.5% crude protein, 24.2% crude fat, 3.2% crude fiber, 5.6% crude ash, 1.2% calcium, and 0.8% total phosphorus). At week 4, mouse serum was collected to detect the levels of four indicators: TG, TC, LDL-C, and HDL-C (Li et al., 2022a; Ge et al., 2022). The treatment group was continuously administered TCDCA (25, 50, and 100 mg/kg/day) (Xu et al., 2023b) and fenofibrate (50 mg/kg/day) (Sohn et al., 2017) by gavage for 4 weeks. The model and control groups were administered the same volume of normal saline.

### 2.4 Efficacy evaluation

After the last dose and fasting for 12 h, the body weight of the mice was recorded. The levels of AST, ALT, TC, TG, LDL-C, and HDL-C in mouse serum were detected again, and the liver index was calculated using the following formula: (wet weight of the liver/mouse body weight) × 100%.

### 2.5 HE staining

After mice were deeply anesthetized with sodium pentobarbital, livers were removed and fixed with 4% paraformaldehyde. After fixation, they were washed with different concentrations of ethanol and water and finally stained with hematoxylin for 5 min. The procedure for staining is as follows: first, the samples were washed with varying concentrations of ethanol, rinsed with water, and stained with hematoxylin for 5 min; then, the samples were differentiated with 1% hydrochloric acid in alcohol for 30 s, rinsed with water for 5 min, and stained with 0.5% eosin solution for 1 min and again rinsed with water for 3 min. Finally, the samples were dehydrated until they became transparent, mounted, and photographed using a microscope (Nikon ECLIPSE Ci-L) (magnification: ×400). The images of the samples were captured.

### 2.6 ORO staining

Fresh liver tissue sections were fixed, washed, and dried. The dried samples were stained with Oil Red O for 10 min, removed, and immersed in 60% isopropanol twice for differentiation and immersed in pure water twice for washing, 10 s each time. The slices were removed and then stained with hematoxylin for 3–5 min. They were immersed in sterile water three times. Subsequently, they were differentiated with 60% ethanol for 8 s and washed with distilled water two times. The slices were washed with hematoxylin blue returning solution to return the blue color for 1 s and washed with sterile water three times. Then, the slices were differentiated with 60% ethanol for 8 s and washed with distilled water two times. The slices were sealed with glycerin, and the images were acquired.

### 2.7 LC-MS metabolomic and lipidomic analyses

#### 2.7.1 Sample preparation

Sample preparation for untargeted metabolomics: Serum or liver tissue homogenate was mixed with acetonitrile (1:3, v/v) and centrifuged at 13,000 rpm for 10 min at 4 °C. The supernatant was evaporated to dryness, and the residue was re-dissolved in acetonitrile–water solution (1:1, v/v). After centrifugation at high speed, the supernatant was collected and assayed. A measure of 2 μL filtrate was injected into the instrument for analysis. Quality control (QC) samples were injected after every seven samples to monitor the reproducibility of the metabolomic analysis results.

Sample preparation for lipid metabolism analysis: Serum or liver tissue homogenate (50 μL) was mixed with ice methanol (200 μL) at a volume ratio of 1:4 and vortexed for 60 s. Ice MTBE (1,000 μL) was added and vortexed for 60 s. Finally, deionized water (200 μL) was added, vortexed for 300 s, and centrifuged at 13,500 r/min for 10 min at 4°C. The upper lipid extract was collected and blown dry under nitrogen at 40°C. The buffer containing internal standards (500 ng each of hexadecanoic acid-d31, d5-TG (16:0/18:0/16:0), d31-PC (16:0/18:1), cholesterol-d7, Cer (d18:1/16:0), and SM (d18:1/17:0)) was passed through 200 μL of isopropanol/acetonitrile (1:1, v/v). The buffer was re-dissolved, and the collected samples were used to perform lipidomic analysis.

#### 2.7.2 UPLC-Orbitrap/MS analysis

Samples were detected using an ultra-high-performance liquid phase (Dionex UltiMate 3000, USA) tandem electrostatic field Orbitrap high-resolution mass spectrometer (Thermo Fisher Orbitrap Fusion, USA). Serum and liver tissue samples were subjected to gradient elution on a Waters ACQUITY UPLC HSS T3 column (1.8 μm, 2.1 × 100 mm) at a 0.4 mL/min flow rate. The ionization source conditions were as follows: 2.8 kV negative ion mode in the ion source type (ESI−), ion transfer tube temperature: 300°C, evaporator temperature: 320°C, sheath gas: 20 Arb, auxiliary gas: 6 Arb, and mass scan range: 100–1000 m/z. Fragment energies of 15%, 25%, and 35%, respectively, were observed, with a resolution of 15,000 and 6 s dynamic reproduction time. The stability of the analysis was continuously monitored by systematically analyzing QC samples every 10 s. The mobile phases included water, 0.1% formic acid (phase A), and acetonitrile (phase B). The elution B phase gradients were as follows: 2% (0–1 min), 2%–35% (1–4 min), 35%–100% (4–13 min), 100%–100% (13–15.5 min), and 100%–2% (15.5–19 min) for metabolomic analysis. The mobile phase consisted of acetonitrile: water (3:2, 10 mM ammonium formate and 0.1% formic acid) (phase A) and isopropanol: acetonitrile (9:1, 10 mM ammonium formate and 0.1% formic acid) (phase B). The elution phase B gradients were as follows: 20% (0 min), 20%–65% (0–4 min), 65%–80% (4–8 min), 80%–95% (8–11 min), 95%–95% (11–12 min), 95%–20% (12–13 min), and 20%–20% (13–15 min) for lipidomic analysis.

#### 2.7.3 Multivariate data analysis

The raw metabolomic data acquired from UHPLC-Orbitrap Fusion MS were treated for peak picking using Orbitrap Fusion Tune and for peak alignment using Xcalibur 4.3 software (Thermo Fisher, USA). We obtained potential and critical information from raw data files via Orbitrap Fusion Tune using Progenesis QI software (Waters, USA). Metabolic data were obtained for health and disease through principal component analysis and dimensionality reduction. Orthogonal partial least squares discriminant analysis (OPLS-DA) was used to determine potential metabolite differences. Objective numerical values screened out the components that significantly impact the grouping.

### 2.8 Statistical analysis

All data are presented as mean ± standard deviation (SD). Data were subjected to *t*-test using GraphPad Prism 7 (GraphPad Software, United States). A *p*-value < 0.05 was considered a significant difference. A *p*-value < 0.01 was supposed to be highly effective. Histograms were drawn using GraphPad Prism 7 software.

### 2.9 Pathway analysis

Results from serum metabolomics were analyzed using MetaboAnalyst (www.metaboanalyst.ca) to find critical metabolic pathways. KEGG (www.kegg.jp) was used to find connections between pathways.

## 3 Results

### 3.1 Body weight and liver index in mice

**FIGURE 1:**
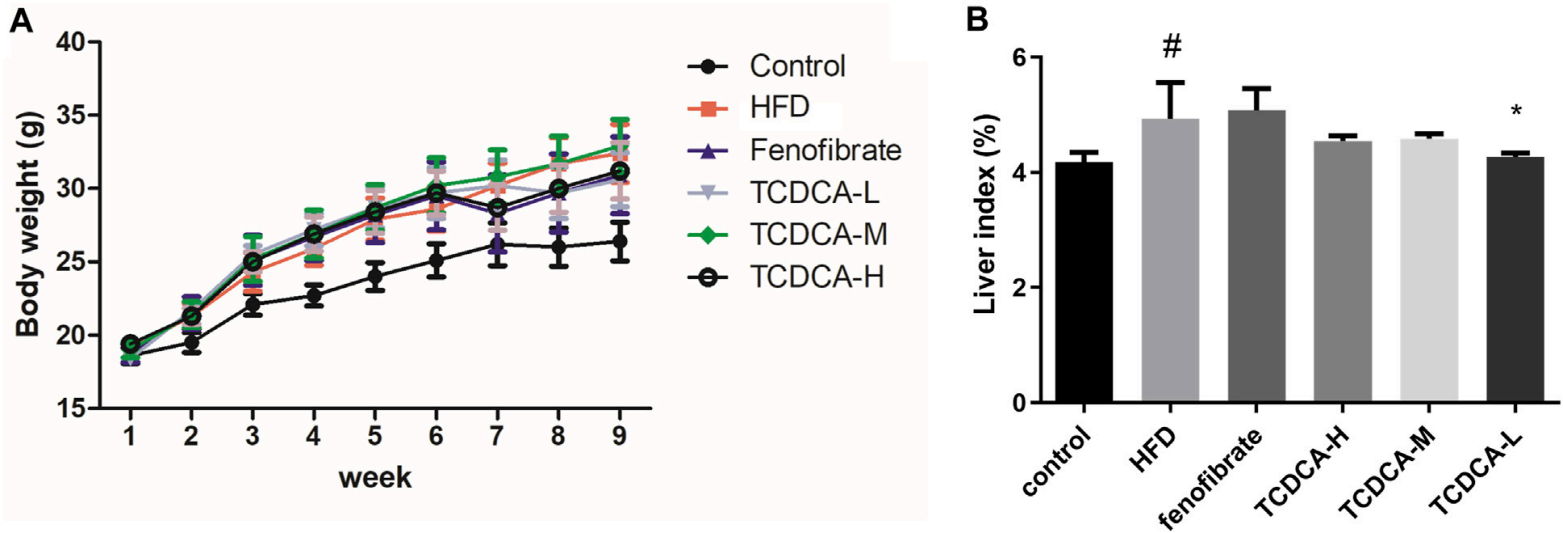
Behavioral indicators of mice in each group: **(A)** body weight and **(B)** liver index. Each value represents the mean ± SD (n = 6). #*p* < 0.05 compared with the control group; ***p < 0.001 and *p < 0.05 compared with the HFD group.

Compared with the control group, the liver index and body weight of mice were significantly increased in the HFD group. Compared with the HFD group, the liver index and body weight of the mice treated with TCDCA-L were significantly reduced (Figure 1).

### 3.2 Physiological and biochemical indicators of mice

**FIGURE 2:**
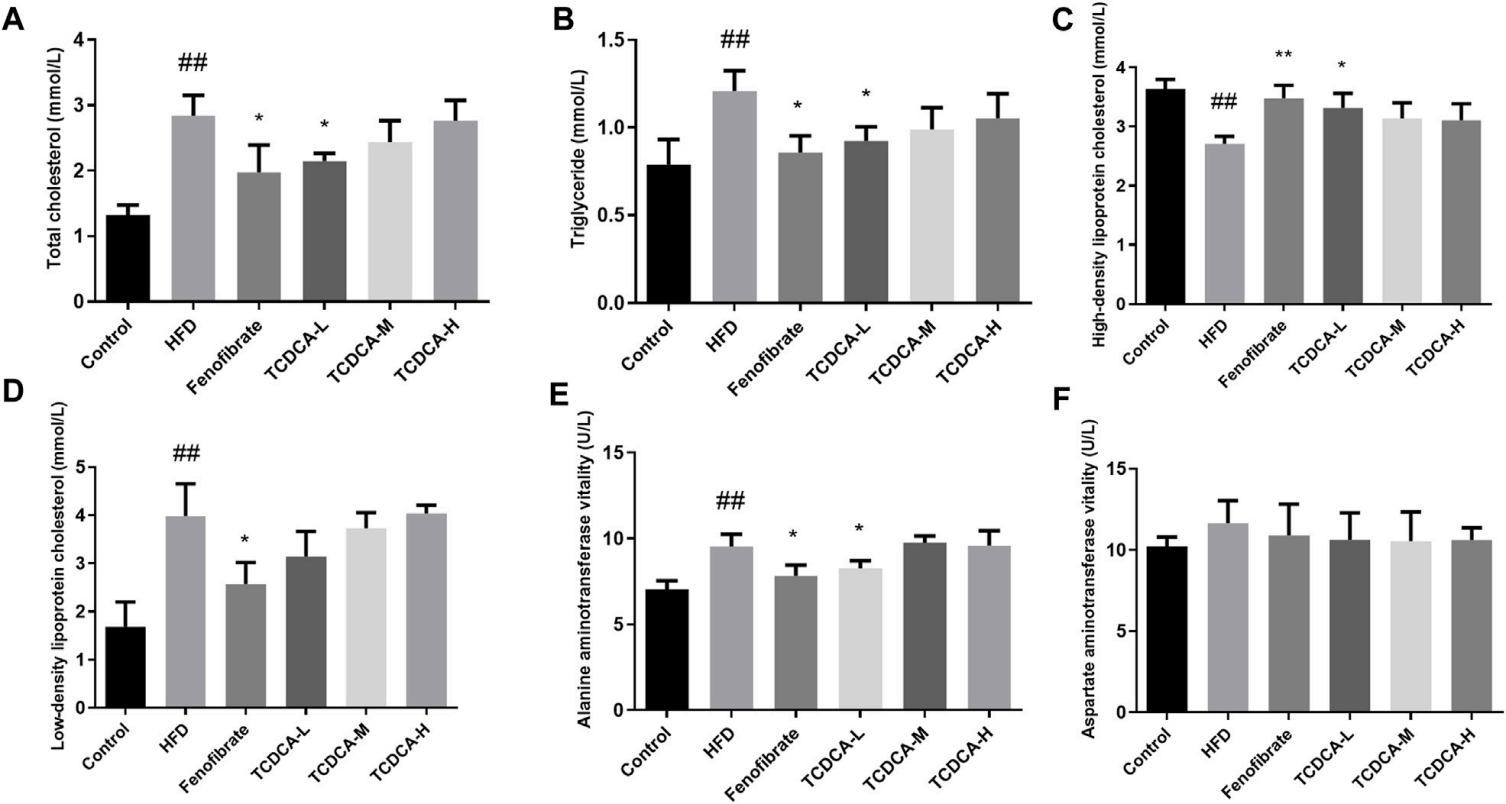
Physiological and biochemical indicators of mice in each group: **(A)** TC, **(B)** TG, **(C)** HDL-C, **(D)** LDL-C, **(E)** ALT vitality, and **(F)** AST vitality. Each value represents the mean ± SD (n = 6). ##*p* < 0.01 compared with the control group; **p < 0.01 and *p < 0.05 compared with the HFD group.

We established a hyperlipidemia mouse model to study the therapeutic effect of TCDCA. During HFD feeding, we observed that the levels of TC, TG, and LDL-C in the blood of the HFD group were significantly increased. At the same time, the concentration of HDL-C was significantly decreased compared to the control group. The dramatic changes in lipid levels in the serum samples of the mice indicated that the hyperlipidemia model was successfully established. Subsequently, different doses of TCDCA and fenofibrate were administered to treat the hyperlipidemia model mice for 30 days, respectively, and we observed that the TCDCA-L group showed a more striking effect compared to the HFD group in which the levels of TC, TG, and HDL-C were significantly downregulated. However, LDL-C levels showed a downward trend with no significant difference. The ALT level in the fenofibrate group was considerably higher than that in the HFD group. There was no significant difference in ALT and AST levels in the other groups (Figure 2).

### 3.3 HE staining

**FIGURE 3:**
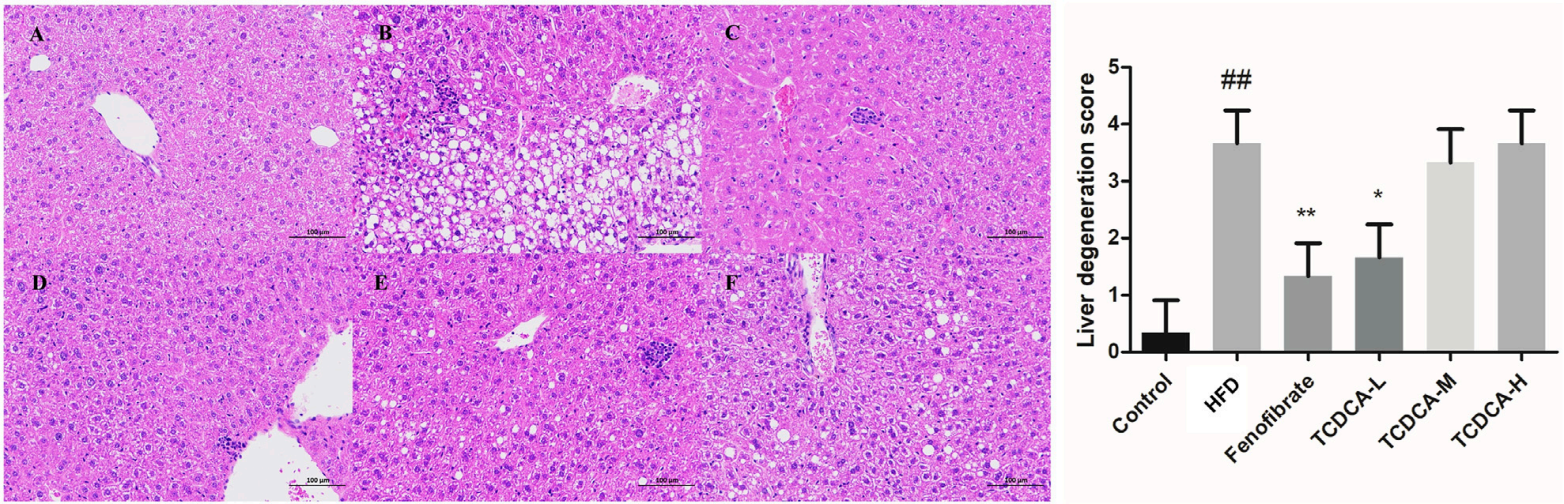
HE staining of hyperlipidemic mouse liver (×400): **(A)** control group, **(B)** HFD group, **(C)** fenofibrate group, **(D)** TCDCA-L group, **(E)** TCDCA-M group, and **(F)** TCDCA-H group.

The changes in the liver tissue of hyperlipidemic mice are shown in Figure 3. HE-stained liver sections of the HFD group showed more rounded lipid droplets and large vesicular steatosis than those of the control group, and the structure of the liver tissue was unclear, with a large number of hepatocytes with granular degeneration, sparse cytoplasm, eosinophilic granules, and a small number of lymphocytes with mildly focal infiltration in the periphery. At the end of the trial, TCDCA-L treatment significantly attenuated fatty liver and lipid deposition, and hepatic lobular and confluent area lesions were relieved.

### 3.4 ORO staining

**FIGURE 4:**
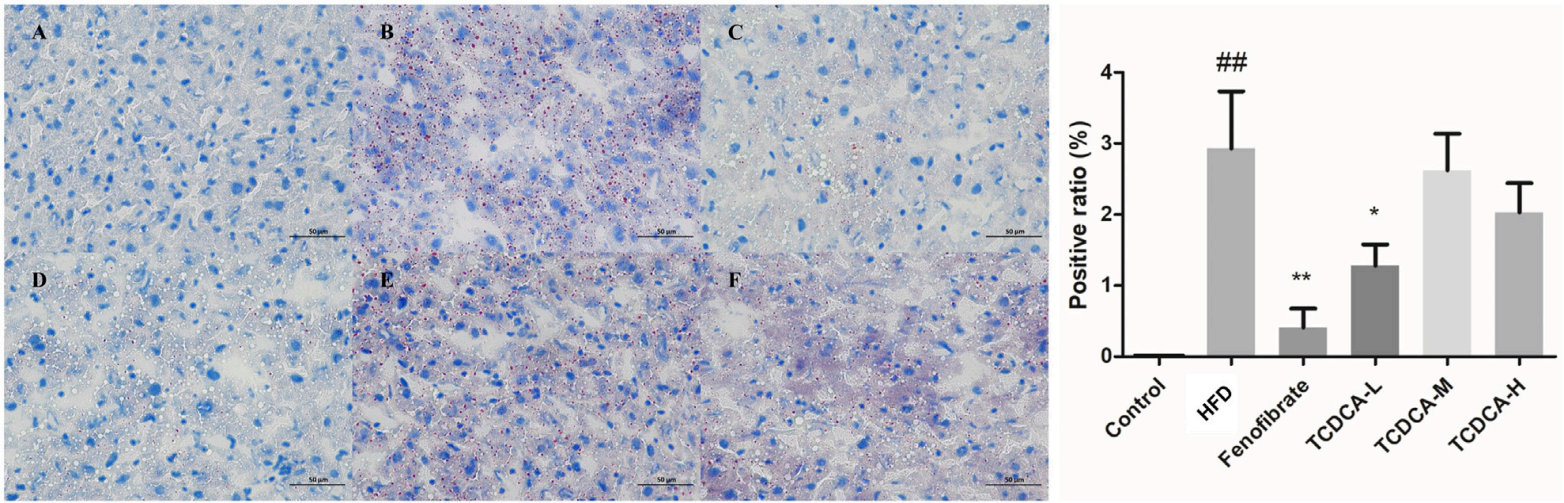
ORO staining of hyperlipidemic mouse liver (×400): **(A)** control group, **(B)** HFD group **(C)** fenofibrate group, **(D)** TCDCA-L group, **(E)** TCDCA-M group, and **(F)** TCDCA-H group.

The hepatocyte deposits of neutral fat were red in ORO staining. At the same time, the control group showed no evident fat deposits, and the HFD group showed more intracellular neutral fat deposits with a more extensive distribution. In the TCDCA group, the deposition of neutral fat in hepatocytes was reduced to varying degrees, and the distribution range was narrowed, especially in the TCDCA-L group (Figure 4).

### 3.5 Results of metabolomic analysis

#### 3.5.1 Multivariate data analysis

**FIGURE 5:**
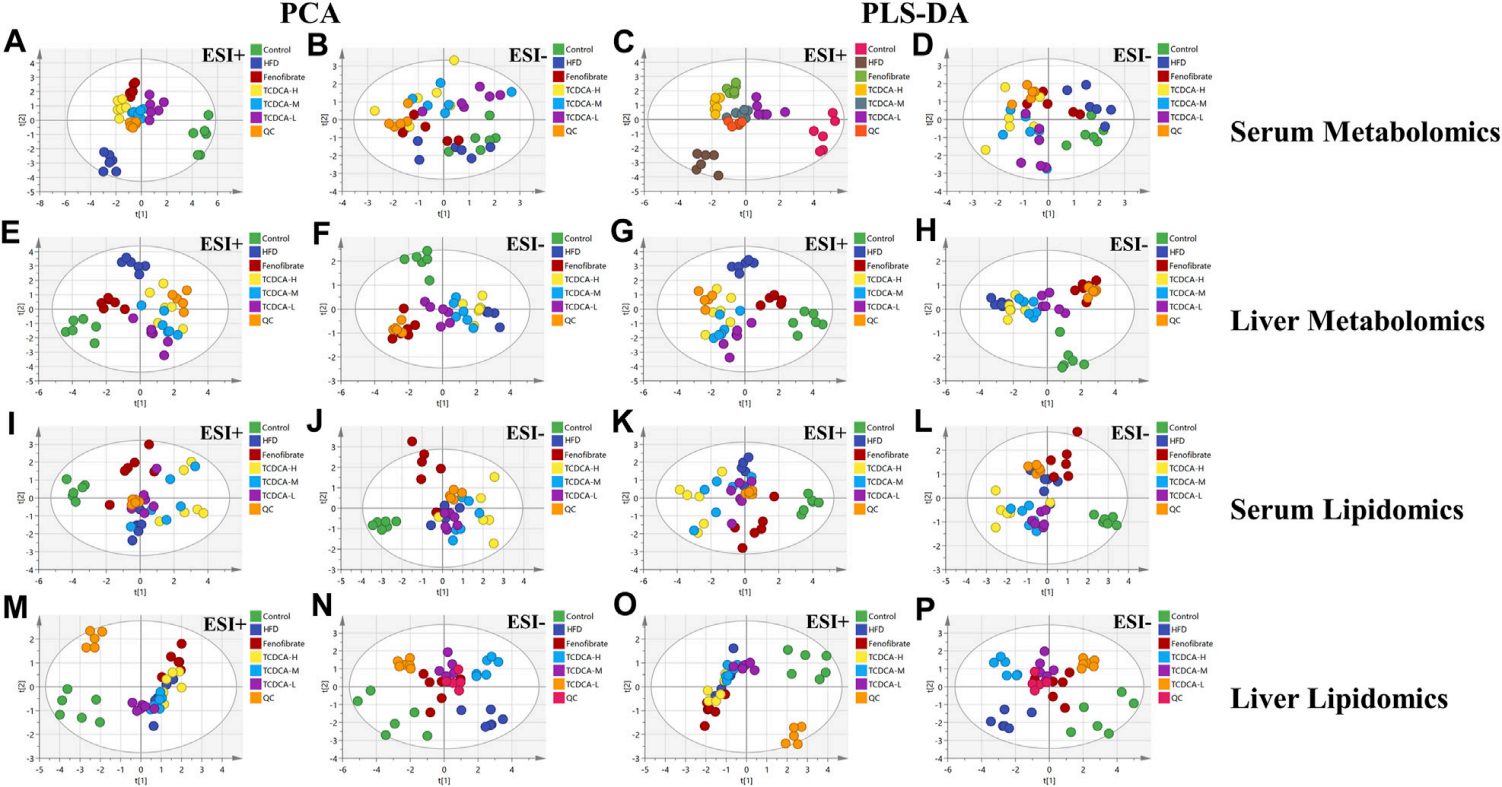
Multivariate data of UPLC-MS/MS: **(A,B)** PCA and **(C,D)** PLS-DA of each group in ESI-negative and -positive ion modes in serum metabolomics; **(E,F)** PCA and **(G,H)** PLS-DA of each group in ESI-positive and -negative ion modes in serum lipidomics; **(I,J)** PCA and **(K,L)** PLS-DA of each group in the ESI-positive and -negative ion modes of liver metabolomics; **(M,N)** PCA and **(O,P)** PLS-DA of each group in ESI-negative and -positive ion modes of liver lipidomics.

In this study, UHPLC-Orbitrap Fusion MS was used for metabolomic analysis. Chromatographic peaks from Orbitrap Fusion Tune raw files were extracted and compared using Xcalibur 4.3 and Progenesis QI software programs. To maximize the collection of metabolomic and lipidomic information and fingerprints of the hyperlipidemia model, unsupervised principal component analysis (PCA) of serum and hepatic metabolites of the six groups (control, HFD, fenofibrate, TCDCA-L, TCDCA-M, and TCDCA-H) was performed in both HESI-positive and HESI-negative modes (Figure 5) The PCA results showed a clear separation between the six groups. The TCDCA group moved toward the control group to varying degrees; particularly, the TCDCA-L group was closer to the control group. The results showed that TCDCA-L had a significant positive effect on hyperlipidemic mice.

To search for potential metabolite markers [variable importance in projection (VIP) > 1, *p* < 0.05], we conducted partial least squares discriminant analysis (PLS-DA). Again, it showed that different groups between the components were more prominent, and there was a significant difference between the control and HFD groups, indicating that the overall metabolic state of the organism changed after 4 weeks. After administration of TCDCA, all positive- and negative-ionized hyperlipidemic mice showed different degrees of improvement, with TCDCA-L showing the most significant performance.

#### 3.5.2 Identification of endogenous metabolites

The differential metabolites between the control group, HFD group, and TCDCA-L group were screened using the VIP value (VIP >1.0) and *t*-test (*p* < 0.05) in metabolomics and lipidomics. A total of 15 metabolites were screened in serum metabolomics (Table 1). Compared with the control group, the model group screened out five metabolites (including PC(18:0/14:0), PI(16:1(9Z)/16:0), and PC(16:0/16:0)) that were significantly increased and six metabolites (including glycocholic acid, 3-hydroxyisovaleric acid, and acetaminophen) that were significantly decreased. Compared with the HFD group, the TCDCA-L group screened out four metabolites (including LysoPC(14:0/0:0), PI(16:1(9Z)/16:0), and hydroxykynurenine) that were significantly increased and four metabolites (including PC(18:0/14:0), sodium glycocholate, and glycocholic acid) that were significantly decreased in serum metabolomics. A total of 13 metabolites were screened in liver metabolomics (Table 2). Compared with the control group, the HFD group screened out four metabolites (including uracil, adenosine, and glycylleucine) that were significantly increased and two metabolites (stachydrine and 5-methoxyindoleacetate) that were significantly decreased. Compared with the HFD group, the TCDCA-L group screened out six metabolites (arachidonic acid, D-tryptophan, and PC(16:0/18:0)) that were significantly increased and two metabolites (5-methoxyindoleacetate and 4-pyridoxic acid) that were significantly decreased.

**TABLE 1:** Detection of hyperlipidemia-related metabolites in serum metabolomics. Trend 1 is the control group compared with the HFD group; Trend 2 is the HFD group compared with the TCDCA-L group.

No.	Metabolite name	TR (min)	m/z	Formula	VIP	*p*-value	FC	Trend 1	Trend 2	Scan mode
1	LysoPC(14:0/0:0)	12.70	490.2901	C_22_H_46_NO_7_P	2.48	3.28 × 10^−3^	1.66	-	↑	+
2	PC(18:0/14:0)	12.85	756.5495	C_40_H_80_NO_8_P	2.20	6.24 × 10^−3^	0.93	↑	↓	+
3	Sodium glycocholate	9.08	488.2978	C_26_H_43_NO_6_	4.08	8.33 × 10^−5^	0.66	-	↓	+
4	Glycocholic acid	9.52	488.2979	C_26_H_43_NO_6_	1.87	1.34 × 10^−2^	1.24	↓	↓	+
5	3-Hydroxyisovaleric acid	10.19	117.0555	C_5_H_10_O_3_	7.64	2.31 × 10^−8^	0.63	↓	↓	-
6	PI(16:1(9Z)/16:0)	12.97	853.5103	C_41_H_77_O_13_P	1.93	1.17 × 10^−2^	0.83	↑	↑	-
7	Hydroxykynurenine	17.13	223.0731	C_10_H_12_N_2_O_4_	8.36	4.33 × 10^−9^	1.38	-	↑	-
8	4-Hydroxynonenal	18.64	215.1143	C_9_H_16_O_2_	7.66	2.19 × 10^−8^	1.72	-	↑	-
9	Acetaminophen	17.29	210.0626	C_8_H_9_NO_2_	4.17	6.82 × 10^−5^	0.93	↓	-	-
10	Acetaminophen glucuronide	3.12	326.0892	C_14_H_17_NO_8_	1.70	2.01 × 10^−2^	1.15	↓	-	-
11	Prostaglandin D1	8.22	353.2335	C_20_H_34_O_5_	3.35	4.49 × 10^−4^	0.32	↑	-	-
12	Threonic acid	0.86	137.0446	C_4_H_8_O_5_	3.90	1.25 × 10^−4^	0.26	↓	-	+
13	PC(16:0/16:0)	8.48	756.5485	C_40_H_80_NO_8_P	1.19	6.42 × 10^−2^	1.39	↑	-	+
14	PI(18:0/22:4(7Z,10Z,13Z,16Z))	15.36	937.5740	C_49_H_87_O_13_P	3.55	2.80 × 10^−4^	3.95	↓	-	+
15	Estrone	3.99	293.1507	C_18_H_22_O_2_	2.34	4.53 × 10^−3^	2.27	↑	-	+

**TABLE 2:** Detection of hyperlipidemia-related metabolites in liver metabolomics. Trend 1 is the control group compared with the HFD group; Trend 2 is the HFD group compared with the TCDCA-L group.

No.	Metabolite name	TR (min)	m/z	Formula	VIP	*p*-value	FC	Trend 1	Trend 2	Scan mode
1	Stachydrine	0.71	143.0952	C_7_H_13_NO_2_	4.97	1.08 × 10^−5^	0.29	↓	-	+
2	Uracil	1.45	112.0279	C_4_H_4_N_2_O_2_	3.86	1.38 × 10^−4^	1.75	↑	-	+
3	Adenosine	0.73	267.0983	C_10_H_13_N_5_O_4_	3.42	3.80 × 10^−4^	3.56	↑	-	+
4	Glycylleucine	3.51	188.1178	C_8_H_16_N_2_O_3_	3.40	4.01 × 10^−4^	7.78	↑	-	+
5	PC(16:0/16:0)	12.84	756.5485	C_40_H_80_NO_8_P	1.16	6.91 × 10^−2^	1.93	↑	-	+
6	5-Methoxyindoleacetate	5.46	205.0733	C_11_H_11_NO_3_	1.43	3.75 × 10^−2^	0.54	↓	↓	-
7	Arachidonic acid	13.00	304.2403	C_20_H_32_O_2_	6.18	6.56 × 10^−7^	0.34	-	↑	-
8	Taurochenodeoxycholic acid	6.34	499.2984	C_26_H_45_NO_6_S	1.58	2.60 × 10^−2^	0.27	-	↑	-
9	D-Tryptophan	3.93	204.0894	C_11_H_12_N_2_O_2_	4.96	1.09 × 10^−5^	0.36	-	↑	-
10	4-Pyridoxic acid	1.84	183.0522	C_8_H_9_NO_4_	7.74	1.83 × 10^−8^	2.27	-	↓	-
11	PC(16:0/18:0)	8.10	761.6008	C_42_H_84_NO_8_P	1.54	2.87 × 10^−2^	0.51	-	↑	-
12	4-Acetamidobutanoic acid	3.20	145.0732	C_6_H_11_NO_3_	1.60	2.49 × 10^−2^	0.53	-	↑	-
13	LysoPC(14:0/0:0)	10.02	490.2901	C_22_H_46_NO_7_P	2.58	2.63 × 10^−3^	1.84	-	↑	+

**FIGURE 6:**
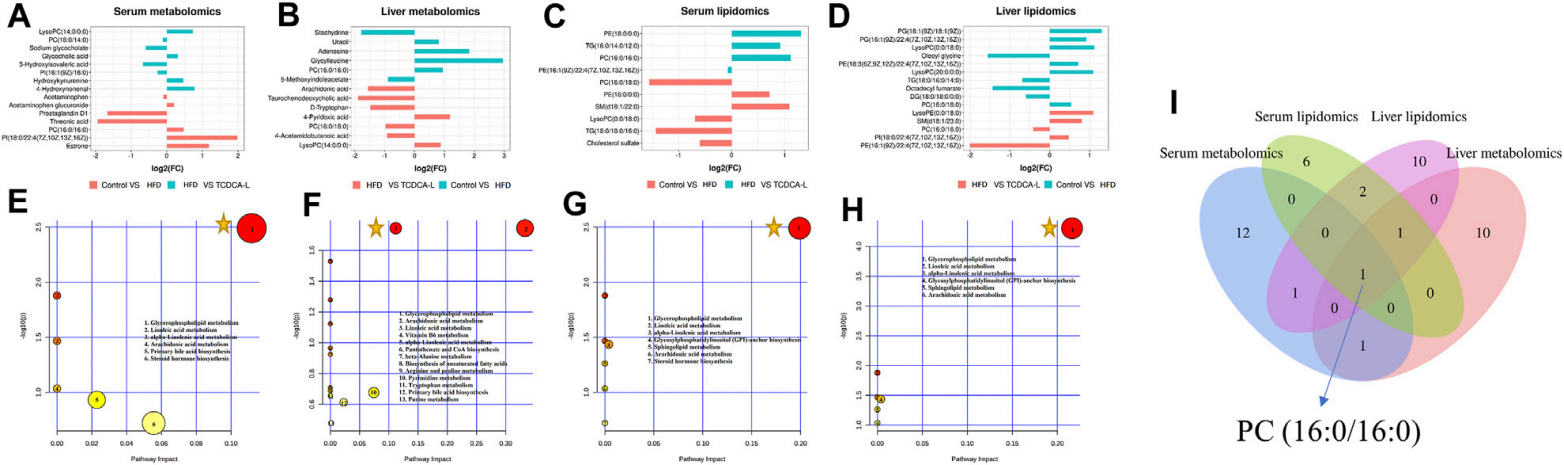
FC value histograms and bubble plots of serum and liver metabolomics and lipidomics: **(A,E)** serum metabolomics; **(B,F)** liver metabolomics; **(C,G)** serum lipidomics; **(D,H)** liver lipidomics; **(I)** Venn diagrams of serum and liver metabolomic and lipidomic metabolites.
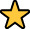
represents an important pathway.

A total of 10 metabolites were screened in serum lipidomics (Table 3). Compared with the control group, the HFD group screened out five metabolites (such as PC(16:0/18:0), SM(d18:1/22:0), and TG(18:0/18:0/16:0)) that were significantly increased and one metabolite (PE(16:0/0:0)) that was significantly decreased. Compared with the HFD group, the TCDCA-L group screened out three metabolites (TG(16:0/14:0/12:0), PC(16:0/16:0), and PE(16:1(9Z)/22:4(7Z,10Z,13Z,16Z))) that were significantly increased and one metabolite (PE(18:0/0:0)) that was significantly decreased. A total of 15 metabolites were screened in liver lipidomics (Table 4). Compared with the control group, the HFD group screened out five metabolites (including PG(18:1(9Z)/18:1(9Z)), PG(16:1(9Z)/22:4(7Z,10Z,13Z,16Z)), and LysoPC(0:0/18:0)) that were significantly increased and four metabolites (including oleoyl glycine, TG(18:0/16:0/14:0), and octadecyl fumarate) that were significantly decreased. Compared with the HFD group, the TCDCA-L group screened out four metabolites (including PC(16:0/18:0), LysoPE(0:0/18:0), and SM(d18:1/23:0)) that were significantly increased and two metabolites (PC(16:0/16:0) and PE(16:1(9Z)/22:4(7Z,10Z,13Z,16Z))) that were significantly decreased. As shown in Figure 6I, metabolomic and lipidomic analyses of serum and liver revealed a common metabolite PC (16:0/16:0).

**TABLE 3:** Detection of hyperlipidemia-related metabolites in serum lipidomics. Trend 1 is the control group compared with the HFD group; Trend 2 is the HFD group compared with the TCDCA-L group.

No.	Metabolite name	TR (min)	m/z	Formula	VIP	*p*-value	FC	Trend 1	Trend 2	Scan mode
1	PE(18:0/0:0)	4.21	481.3153	C_23_H_48_NO_7_P	2.02	9.64 × 10^−3^	2.48	-	↓	-
2	TG(16:0/14:0/12:0)	11.28	739.6759	C_45_H_86_O_6_	1.64	2.31 × 10^−2^	1.89	-	↑	+
3	PC(16:0/16:0)	7.39	733.5681	C_40_H_80_NO_8_P	2.00	9.97 × 10^−3^	2.17	-	↑	+
4	PE(16:1(9Z)/22:4(7Z,10Z,13Z,16Z))	3.14	765.5312	C_43_H_76_NO_8_P	3.52	3.03 × 10^−4^	0.95	-	↑	-
5	PC(16:0/18:0)	8.02	761.5996	C_42_H_84_NO_8_P	5.90	1.26 × 10^−6^	0.34	↑	-	+
6	PE(16:0/0:0)	3.10	453.2840	C_21_H_44_NO_7_P	1.36	4.35 × 10^−2^	1.64	↓	-	-
7	SM(d18:1/22:0)	8.75	832.6645	C_45_H_91_N_2_O_6_P	12.58	2.61 × 10^−13^	2.13	↑	-	-
8	LysoPC(0:0/18:0)	3.87	523.3683	C_26_H_54_NO_7_P	2.67	2.13 × 10^−3^	0.62	↑	-	+
9	TG(18:0/18:0/16:0)	12.94	879.8329	C_55_H_106_O_6_	2.61	2.47 × 10^−3^	0.37	↑	-	+
10	Cholesterol sulfate	5.02	466.3101	C_27_H_46_O_4_S	7.05	8.83 × 10^−8^	0.66	↑	-	-

**TABLE 4:** Detection of hyperlipidemia-related metabolites in liver lipidomics. Trend 1 is the control group compared with the HFD group; Trend 2 is the HFD group compared with the TCDCA-L group.

No.	Metabolite name	TR (min)	m/z	Formula	VIP	*p*-value	FC	Trend 1	Trend 2	Scan mode
1	PG(18:1(9Z)/18:1(9Z))	6.57	774.5369	C_42_H_79_O_10_P	8.22	5.98 × 10^−9^	2.48	↑	-	-
2	PG(16:1(9Z)/22:4(7Z,10Z,13Z,16Z))	6.15	796.5219	C_44_H_77_O_10_P	6.67	2.13 × 10^−7^	1.89	↑	-	-
3	LysoPC(0:0/18:0)	4.05	569.3664	C_26_H_54_NO_7_P	4.23	5.85 × 10^−5^	2.17	↑	-	-
4	Oleoyl glycine	1.43	339.2782	C_20_H_37_NO_3_	4.09	8.19 × 10^−5^	0.34	↓	-	+
5	PE(18:3(6Z,9Z,12Z)/22:4(7Z,10Z,13Z,16Z))	6.89	789.528	C_45_H_76_NO_8_P	3.95	1.11 × 10^−4^	1.64	↑	-	-
6	LysoPC(20:0/0:0)	5.23	551.4011	C_28_H_58_NO_7_P	3.15	7.10 × 10^−4^	2.13	↑	-	+
7	TG(18:0/16:0/14:0)	12.38	823.7714	C_51_H_98_O_6_	3.06	8.66 × 10^−4^	0.62	↓	-	+
8	Octadecyl fumarate	4.91	368.2939	C_22_H_40_O_4_	2.75	1.78 × 10^−3^	0.37	↓	-	+
9	DG(18:0/18:0/0:0)	10.21	641.6022	C_39_H_76_O_5_	2.12	7.50 × 10^−3^	0.66	↓	-	+
10	PC(16:0/18:0)	7.88	807.5948	C_42_H_84_NO_8_P	2.05	8.89 × 10^−3^	1.45	-	↑	-
11	LysoPE(0:0/18:0)	4.18	481.3144	C_23_H_48_NO_7_P	1.93	1.18 × 10^−2^	2.13	-	↑	-
12	SM(d18:1/23:0)	8.9	846.6783	C_46_H_93_N_2_O_6_P	1.46	3.50 × 10^−2^	1.74	-	↑	-
13	PC(16:0/16:0)	7.57	733.57	C_40_H_80_NO_8_P	1.37	4.27 × 10^−2^	0.75	-	↓	+
14	PI(18:0/22:4(7Z,10Z,13Z,16Z))	7.17	914.5831	C_49_H_87_O_13_P	1.36	4.36 × 10^−2^	1.39	-	↑	-
15	PE(16:1(9Z)/22:4(7Z,10Z,13Z,16Z))	7.14	765.5273	C_43_H_76_NO_8_P	2.21	6.22 × 10^−3^	0.25	-	↓	-

#### 3.5.3 Analysis of metabolic pathways

To further understand the metabolic pathways in TCDCA-treated mice, metabolic pathway analyses of different metabolites enriched were performed using MetaboAnalyst 5.0 (www.MetaboAnalyst. ca). In liver and serum metabolomics and lipidomics, to compare the expression levels of metabolites between samples, based on fold-change values, we created FC value histograms (Figures 6A; 6B; 6C; 6D) and input metabolites into MetaboAnalyst to explore the metabolic pathways of TCDCA in the treatment of hyperlipidemia. The results showed that TCDCA might affect the glycerophospholipid metabolism and has the most significant impact, proving that it is one of the essential metabolic pathways (Figures 6E–H). The Venn diagram showed one common differential metabolite PC (16:0/16:0) in the four groups of serum, liver, serum lipids, and liver lipids (Figure 6I).

#### 3.5.4 Correlation analysis

**FIGURE 7:**
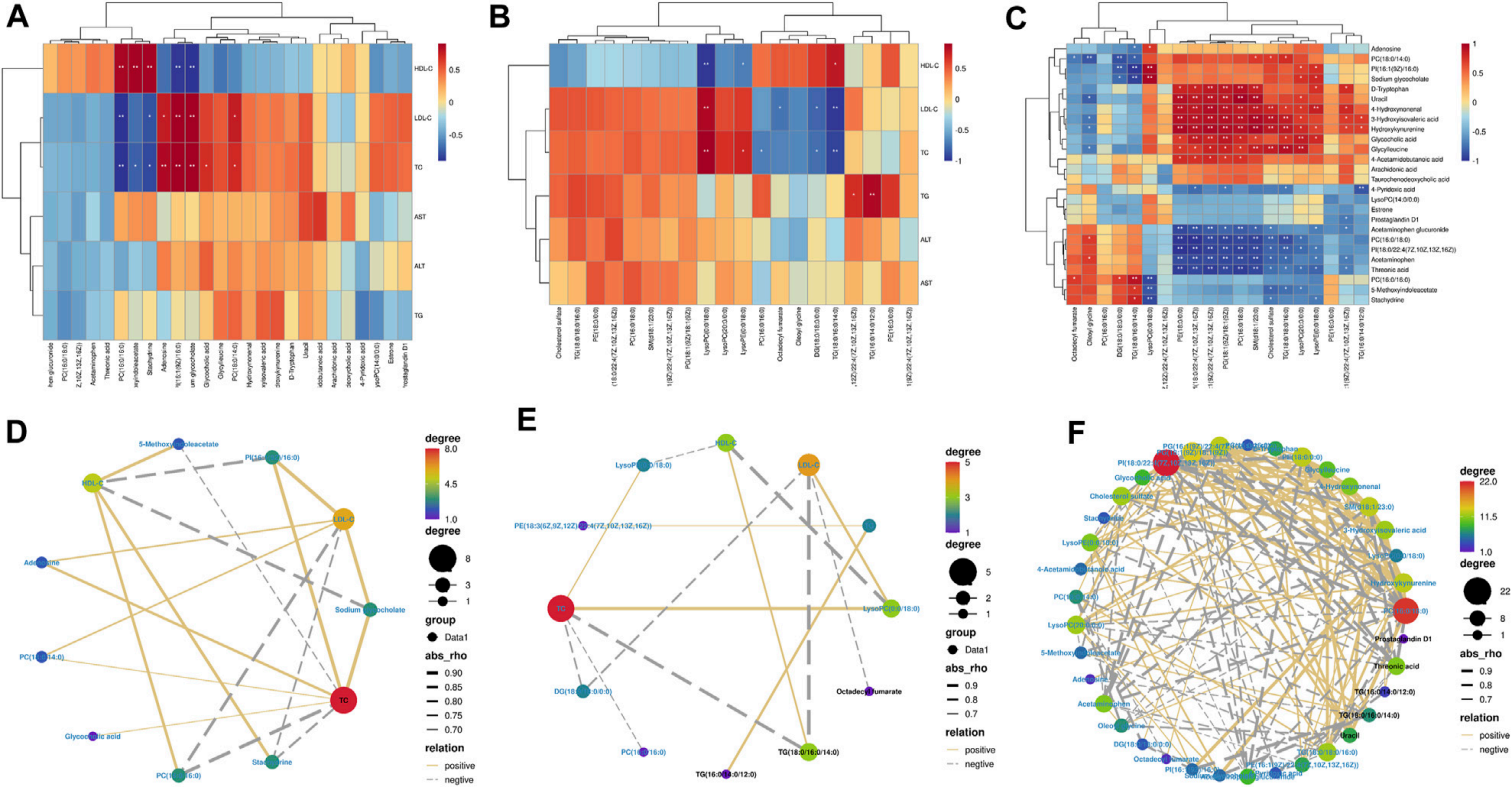
Spearman’s correlation analysis: **(A,D)** serum biochemical indexes with serum and liver metabolomic endogenous metabolites. **(B,E)** Serum biochemical indexes with serum and liver lipidomic endogenous metabolites. **(C,F)** Serum and liver metabolomic endogenous metabolites with serum and liver lipidomic endogenous metabolites.

We performed Spearman’s correlation tests to explore the relationship between metabolites and physiological characteristics. A total of 15 metabolites were correlated with physiological traits associated with hyperlipidemia (HDL-C, LDL-C, TC, TG, ALT, and AST; Figure 7A). Among these 15 metabolites, we observed stronger relationships between metabolites and lipid indicators (HDL-C, LDL-C, and TC) than liver parameters (TG, AST, and ALT). Four metabolites were positively correlated with LDL-C and TC, three metabolites were negatively correlated with LDL-C and TC and negatively correlated with HDL-C; three metabolites were positively correlated with HDL-C; and two metabolites were negatively correlated with HDL-C. For example, PI (16:1(9Z)/16:0) was significantly positively correlated with LDL-C and TC and negatively correlated with HDL-C. A total of 20 lipid metabolites were correlated with physiological characteristics associated with hyperlipidemia (HDL-C, LDL-C, TC, TG, ALT, and AST; Figure 7B) for correlation analysis. Metabolites and hyperlipidemia indicators were analyzed in a correlation network (positive correlation threshold ≥0.5, negative correlation threshold ≤0.5, and a *p*-value threshold ≤0.05; Figure 7D). Among these eight lipid metabolites, we observed stronger relationships between lipid metabolites and lipid metrics (HDL-C, LDL-C, TC, and TG) than liver parameters (AST and ALT). Four metabolites were positively correlated with LDL-C, TC, and TG, four metabolites were negatively associated with LDL-C, TC and TG; one metabolite was positively correlated with HDL-C; two metabolites were positively correlated with HDL-C; and two metabolites were negatively correlated with HDL-C. For example, LysoPC (0:0/18:0) had a significant positive correlation with LDL-C and TC and a significant negative correlation with HDL-C. Lipid metabolites and hyperlipidemia indicators were analyzed in a correlation network (positive correlation threshold ≥0.5, negative correlation threshold ≤0.5, and a *p*-value threshold ≤0.05; Figure 7E). Finally, a correlation analysis was performed between metabolites and lipid metabolites (Figure 7C). Lipid metabolites and metabolite indicators were analyzed in a correlation network (positive correlation threshold ≥0.5, negative correlation threshold ≤0.5, and a *p*-value threshold ≤0.05; Figure 7F).

## 4 Discussion

The pathogenesis of hyperlipidemia is characterized by metabolic dysfunction and abnormal lipid metabolism *in vivo* (Kim and Scherer, 2021). By identifying pathological metabolic changes, metabolomic approaches point to common endogenous markers of diet-induced hyperlipidemia (Xu et al., 2023c). After pharmacological intervention, target differential metabolites are sought. It can be used for early disease diagnosis (Zhang et al., 2012), drug target discovery (Rabinowitz et al., 2011), disease mechanism studies, and disease diagnosis (Beyoğlu and Idle, 2020).

Many studies have confirmed that bile acids promote lipid digestion and absorption, inhibit cholesterol precipitation in bile, reduce cholesterol saturation, and prevent the formation of gallstones (de Aguiar Vallim et al., 2013; Zhou et al., 2023). Bile acids affect cholesterol levels by influencing lipid metabolism in the liver and intestines and also promote the expression of the LDL receptor, which helps lower blood LDL levels. In some patients with hyperlipidemia, bile acids may be used to regulate lipid metabolism and lower cholesterol levels. Chenodeoxycholic acid tablets have been used as gallstone solubilizers to inhibit cholesterol supersaturation, limit hydroxymethylglutaryl coenzyme activity, reduce cholesterol synthesis and secretion, and prevent and dissolve gallstones (Ahlberg et al., 1981). In the studies of animals modeling lipid disorders, drug efficacy was accompanied by an increased abundance of some bile acids, including TUDCA, TCDCA, and CDCA (Li et al., 2022b), and it may be that these increased bile acids enhanced the efficacy of the drugs.

A high-fat diet (HFD) affects the gut microbiota of mice, and changes in gut microbiota structure may affect serum and liver lipids (Kim et al., 2012). In the present study, it was found that in the course of daily dietary HFD in mice, the weight gain after the administration was significantly slower in terms of the mice’s behavior than that in the HFD group. From the four clinical indicators of TC, TG, LDL-C, and HDL-C (Gong et al., 2016), the TCDCA-L group all had a callback effect. Meanwhile, there was no significant difference in the levels of TC, TG, LDL-C, and HDL-C in the TCDCA-H group, but the efficacy was more pronounced in the TCDCA-M group than in the TCDCA-H group. Another symptom of hyperlipidemia is liver enlargement (Bay et al., 2017), which may be caused by excessive fat intake and disturbance of lipid metabolism (Xenoulis and Steiner, 2010). The HE and ORO staining results also demonstrated that TCDCA-L could significantly reduce granular hepatocyte deformation and neutral fat deposition. Therefore, TCDCA was shown to have a lipid-lowering effect.

The liver is the center of lipid metabolism and secretes various enzymes related to lipid metabolism (Wu et al., 2021). In hyperlipidemia, the liver may synthesize excess VLDL, which contains more glycerophospholipids, leading to elevated levels of triacylglycerol and cholesterol in the blood (Tsuji et al., 2021). In addition, the main carrier of cholesterol is low-density lipoprotein (LDL), which transports cholesterol from the body to individual cells (Du et al., 2022). However, abnormalities in glycerophospholipid metabolism caused by hyperlipidemia affect the structure and stability of LDL, which may lead to oxidation or glycosylation of LDL, which in turn creates a burden for cholesterol transport and makes it more likely to accumulate in the lining of the blood vessels, leading to cardiovascular diseases such as atherosclerosis (hardening of the arteries) (Morita, 2016).

Glycerophospholipid metabolism is the common pathway. The key metabolite of this pathway is PC. Previous studies have shown that PC is an important signaling molecule in atherosclerosis and an essential component of cell membranes (Zeng et al., 2017). PC enhances the assembly of VLDL in hepatocytes and the production of bile acids in the liver, thereby promoting lipid export and regulating lipid metabolism (Cole et al., 2012). Therefore, in the present study, we noted the metabolic characteristics of liver and hepatic lipids while observing the changes in serum lipids. The analysis of metabolomic and lipidomic results showed that the efficacy of the TCDCA-L group was higher than that of the other two groups. By enrichment analysis of differential metabolites, we found that TCDCA achieves beneficial effects and regulates glycerophospholipid metabolism by modulating metabolites. Therefore, we concluded that the critical pathway of TCDCA in treating hyperlipidemia may be glycerophospholipid metabolism.

## 5 Conclusion

Our data suggest that TCDCA ameliorates diet-induced hyperlipidemia in mice. Its mechanism may be used to regulate lipid metabolism pathways such as glycerophospholipid metabolism. These findings indicate that TCDCA may be an effective alternative for hyperlipidemia treatment.

## Data Availability

The raw data supporting the conclusion of this article will be made available by the authors, without undue reservation.

## Abbreviations

SD, standard deviation; TCDCA, taurochenodeoxycholic acid; TG, triglycerides; TC, total cholesterol; LDL-C, low-density lipoprotein cholesterol; HDL-C, high-density lipoprotein cholesterol; KEGG, Kyoto Encyclopedia of Genes and Genomes; VIP, variable importance in projection; AD, Alzheimer’s disease; PD, Parkinson’s disease; HD, Huntington’s disease; QC, quality control.
